# Return to work of major trauma survivors from a private level 1 trauma centre in South Africa

**DOI:** 10.1007/s00068-024-02712-7

**Published:** 2025-01-15

**Authors:** Francesca Bharath, Susan Hanekom, Tonya Estherhuizen, Alison Lupton-Smith

**Affiliations:** 1 Division of Physiotherapy, Department of Health and Rehabilitation Sciences, Faculty of Medicine and Health Sciences, Stellenbosch University, Cape Town, South Africa; 2 Division of Epidemiology and Biostatistics, Faculty of Medicine and Health Sciences, Stellenbosch University, Cape Town, South Africa

**Keywords:** Return to work (RTW), Major trauma, Trauma intensive care unit (TICU), Level 1 trauma centre, Chelsea critical care physical assessment (CPAx)

## Abstract

**Purpose:**

Trauma is known as a leading cause of mortality and injury related disability globally. In South Africa (SA) the socioeconomic burden of trauma is magnified as the working age is most affected. The aim of this study was to describe the proportion of major trauma survivors who returned to work (RTW) during a 6-month period post hospital discharge and to identify the factors associated with the RTW outcome.

**Methods:**

This was a prospective observational cohort study involving major trauma survivors from a private level 1 trauma centre intensive care unit in SA between January and September 2022. RTW status was assessed using the Employment Questionnaire. Univariate and multivariable Cox proportional hazards regression was used in analysis.

**Results:**

Sixty-four of the 86 participants (74.4%) RTW at six months post hospital discharge. RTW had a median time of 16 weeks. After adjusting and backwards analysis, Chelsea Critical Care Physical assessment tool scores (adjusted hazard ratio (AHR), 1.06, 95% CI 1.01–1.10, *p* = 0.007), and not having applied/received any form of grants (AHR 2.26, 95% CI 1.35–3.77, *p* = 0.002) were the only factors that were associated with the RTW outcome.

**Conclusion:**

The cumulative probability of no RTW was 25.6% among participants after 24 weeks. Higher physical function at ICU discharge and not seeking any form of compensation was associated with a higher probability of RTW. This study has highlighted the complexities of RTW and the socioeconomic burden following major trauma. There is therefore a need for further studies on RTW following major trauma in SA.

## Introduction

Trauma represents a significant burden on global health [1]. In high-income countries (HICs), trauma is the leading cause of death in people younger than 40 years of age and in low- and middle-income countries (LMICs) it is a neglected epidemic which causes more than five million deaths per year [2, 3]. This is problematic because it is estimated that for every mortality there are between 10 and 50 times more injured survivors, half of whom will have some form of disability [4]. Among trauma cases, major trauma stands out as a leading cause of admissions to intensive care units (ICUs) [5, 6]. Major trauma can be considered as having an injury severity score (ISS) of more than 12, requiring urgent surgery, or being admitted to ICU for more than 24 h [7–9]. In South Africa (SA), an upper-middle-income country (UMIC), trauma presents a significant public health challenge as it is a leading cause of unnatural death and disability within the country [10, 11]. Particularly noteworthy is the high prevalence of major trauma among working-age individuals, predominantly males, in SA [12–14]. This demographic constitutes a substantial portion of the workforce, intensifying the socioeconomic impact of major trauma and imposing considerable costs on society [12–14].

Understanding the long-term outcomes following major trauma survival, particularly return to work (RTW), is crucial. RTW serves as an indicator of functional recovery and represents a vital rehabilitation goal post-major trauma [15, 16]. Delayed RTW not only affects the economy but also diminishes an individual's quality of life [17]. No RTW further exacerbates the burden of major trauma due to ensuing personal, financial, and social implications [7]. RTW rates among major trauma survivors vary between 50 and 70% [18], with studies predominantly conducted in HICs [8, 19–21]. These studies have identified various factors associated with the RTW outcome. Older age [7, 8, 22], presence of co-morbidities [7, 8, 22], higher injury severity scores (ISS) [19, 20, 22], longer hospital length of stay (HLOS) [22], discharge destination other than home [22], receiving compensation [7, 8, 21], physically demanding occupations [7, 8, 22], and mechanism of injury such as from motor vehicle accidents (MVA) [7] are all factors that have been found to have a decreased likelihood of RTW. Despite differences in study designs and contexts, findings across HICs have been largely consistent [7, 8, 19–21]. However, the applicability of these findings to UMICs like SA remains uncertain.

Recent UMIC research, although not specific to the major trauma population, conducted in Botswana, reported an 84% RTW rate post MVAs [23]. This study highlighted the influence of injury severity and the presence of RTW rehabilitation or plans on RTW rates. However, one should also consider that driving factors for RTW such as job security concerns and dependency on an income, which are unique to LMICs, may have contributed to the high rate of RTW found [23]. In SA, research on RTW following major trauma is sparse. Van Aartsen & Van Aswegen [24] reported a 55% RTW rate at six months from a small study conducted on a mixed cohort of ICU survivors who were mechanically ventilated for more than 24 h [24]. Another study focused on lower limb long bone fractures found a 45.1% RTW rate at the same time point [25]. While informative, these studies were not specific to the major trauma population. Nevertheless, they offer some insights into RTW following hospitalisation in SA, albeit with lower RTW rates compared to HICs.

In addition to recovery time and injury severity, socioeconomic factors could possibly play a crucial role in RTW outcomes within the South African context. High unemployment rates, poverty, lower educational levels, and cultural beliefs could significantly influence RTW [26–28]. Disparities within the two-tiered SA healthcare system, with limited access to rehabilitation facilities in the public sector, further compound these challenges [29, 30]. Given these complexities, we chose to conduct our study within a private level 1 trauma centre. This decision was driven by the facility's well-resourced environment, adherence to international standards of care, and access to an employed population, considering the high costs associated with private sector healthcare [27, 31].

Thus, our study aimed to explore RTW outcomes in the SA setting, specifically describing the proportion of major trauma survivors who RTW within a 6-month period post-hospital discharge from a private level one trauma centre. Additionally, we sought to identify factors influencing the RTW outcome in this population.

## Methods

This study is reported according to the Strengthening the Reporting of Observational Studies Guidelines: The STROBE checklist [32]. Ethical approval was obtained from Stellenbosch University, Ethics reference number: S21/04/061 and the board of the Private Hospital Group, approval number: UNIIV-2021-0052. Written informed consent was obtained from all participants prior to enrolment into this study. All participants were assigned a unique identifier when entering the study and all data collected was anonymised prior to the transfer from the data collection form to analysis software.

### Study design

This was a prospective observational cohort study of trauma intensive care unit (TICU) survivors from a private level 1 trauma centre in SA.

### Study setting

Participants were recruited from the TICU of a Private Hospital within the Ekurhuleni metropolitan municipality in Gauteng. This municipality covers an area of 1975 square kilometres and has a population size of just under 3.2 million people. The availability of Helicopter Emergency Medical Services further increases the catchment area, with many major trauma patients being transported from all over Gauteng and neighbouring African countries. This hospital is one of the four Trauma Society of South Africa accredited level one trauma centres in the private sector of SA. The TICU of this level one trauma centre has 24 beds and is run by a team of dedicated trauma surgeons, who then refer to the intensivist, and other specialist doctors as needed. Patients are screened by the dedicated outsourced multidisciplinary trauma team and treated as needed. Patients are usually seen daily or bi-daily as needed by the doctors, physiotherapists, dieticians, and psychologists; as well as by occupational therapists, speech therapists, and social workers as required, all of whom form part of the dedicated team that provide care for major trauma patients within this hospital.

### Enrolment procedure

All patients who were discharged from TICU between January 2022 and September 2022 were approached to participate in this study. Patients were eligible for inclusion if they met the following criteria: patients of both sexes who were of working age (18–64), participating in paid or unpaid work, who had sustained major trauma and had been discharged from TICU were eligible for recruitment. Major trauma was defined by one or more of the following: ISS of more than 12, urgent surgery, or admission to intensive care for more than 24 h following trauma [9]. Patients with the following conditions were not considered for participation in this study: those with final stages of terminal illness or end stage disease, patients who were admitted to ICU specifically for cardiac, medical, surgical or neurological conditions without having sustained major trauma as part of their admitting diagnosis, severe head injuries which resulted in cognitive impairments that impeded participation (determined by a Glascow coma score (GCS) of less than 15/15 and a S5Q score of less than 5/5 at time of ICU discharge) and unable to speak English or Afrikaans or isiZulu in order to provide informed consent.

### Data collection

The primary outcome of RTW status was assessed using the standardised Employment Questionnaire [33, 34]. This questionnaire has been used in prior studies to determine RTW outcomes in the critically ill population and following traumatic head injury [33, 34]. Derived from Collie et al. [7] RTW was defined as returning to any paid or unpaid work in any capacity (full time or part-time) or type of work (same/different job; same/different employer) within the 6-month period prior to follow up interview [7]. All initial in person interviews at ICU discharge for baseline employment status and 6-month follow up telephonic interviews for the current employment status were conducted by the primary researcher or by either the Afrikaans or isiZulu research assistants. A self-developed sociodemographic questionnaire was also administered at the initial interview to obtain the following information: sex, highest educational level, prior disabilities, and presence of co-morbidities. The patient’s recorded Medical Research Sum Score (MRC-SS) and Chelsea Critical Care Physical Assessment tool (CPAx) score at ICU discharge were taken from the patients’ records. These two outcome measures are administered routinely by the treating physiotherapists for all patients in the TICU of this hospital. The CPAx is a validated and reliable, graphical, and numeric tool, which is used to assess physical function in the ICU setting [35]. The CPAx assesses 10 components of physical function: respiratory function, cough, bed mobility, supine to sitting on the edge of the bed, dynamic sitting, sit to stand, standing balance, transferring from bed to chair, stepping and grip strength, and each component is graded from complete dependence to independence [35, 36]. The MRC-SS is a muscle strength test that is most frequently used in critical care research to assess for muscle weakness [37, 38]. The MRC-SS is scored out of 60 and is based on the manual testing of six muscle groups of the upper and lower limbs [39, 40]. The MRC-SS has shown good inter-rater reliability and good inter-observer agreement in the classification of severe weakness [41]. The muscle weakness among ICU patients is known as ICU-Acquired Weakness [42]. A score of less than 48 on the MRC-SS is indicative of ICU-AW [37]. Hospital data sheets was used to obtain information such as age, language, employment history and funding profile. Medibank [Verticalapps, Johannesburg, South Africa] was used at hospital discharge to extract injury related and clinical information such as HLOS, ISS, GCS, mechanism of injury, incidents, and discharge destination. Medibank is a cloud-based software which is used by this Private Hospital Group to track patients by recording their clinical information from an incident to emergency department, to ICU and through to discharge.

Two pilot studies were conducted prior to the main study. Firstly, for standardisation among researcher and research assistants and to check for understanding of the questionnaires both self-developed and Employment Questionnaire among SA participants. All questions were understood by all participants and no changes needed to be made to the tools used. Secondly, to establish inter-rater reliability between the researcher and physiotherapists administering the MRC-SS and CPAx. Excellent inter-rater reliability for MRC-SS (interclass correlation coefficient, ICC = 0.98; 95% confidence interval, CI = 0.92–1) and for CPAx (ICC = 0.92; 95% CI = 0.45–0.99) was demonstrated, and no changes to the study procedure were required. All participant data used in the pilot studies was excluded from the main study analysis.

### Sample size

This study was the first of its kind in SA and given that SA has no national trauma registry or database to draw from as were used by many international studies, an accurate sample size could not be obtained. We, therefore, aimed to collect data from as many participants as possible within a 9-month period, January to September 2022.

### Data analysis

Data analysis was performed in collaboration with the Biostatistics Unit, Division of Epidemiology and Biostatistics, Faculty of Medicine and Health Sciences, Stellenbosch University. All data was analysed using IBM SPSS version 28 [IBM, Armonk, NY, USA]. Normality of continuous variables was tested using the Shapiro–Wilk test. All descriptive statistics that were normally distributed data are presented as means and standard deviations (SD). All non-normally distributed variables are presented as median and interquartile range (IQR) for continuous variables and number (percentage) for categorical variables. Independent *T*-test was used for testing normally distributed data and Mann–Whitney *U* test was used for non-normally distributed numerical data. For testing categorical variables, a chi-square test or fisher’s exact test was used as applicable (both two sided). Statistical significance was evaluated at 5% significance level (*p* < 0.05). For associations of the RTW outcome, all variables with *p* < 0.25 in the initial testing as mentioned above were used in the univariate and multivariable Cox proportional hazards regression analysis. A Kaplan–Meier survival curve was generated for weeks to RTW. Those that had not RTW by the 6-month follow up were censored and given a time to RTW as 25 weeks and a RTW status as no. No assumptions were made for missing data.

## Results

### Overview of participants

**Fig. 1:**
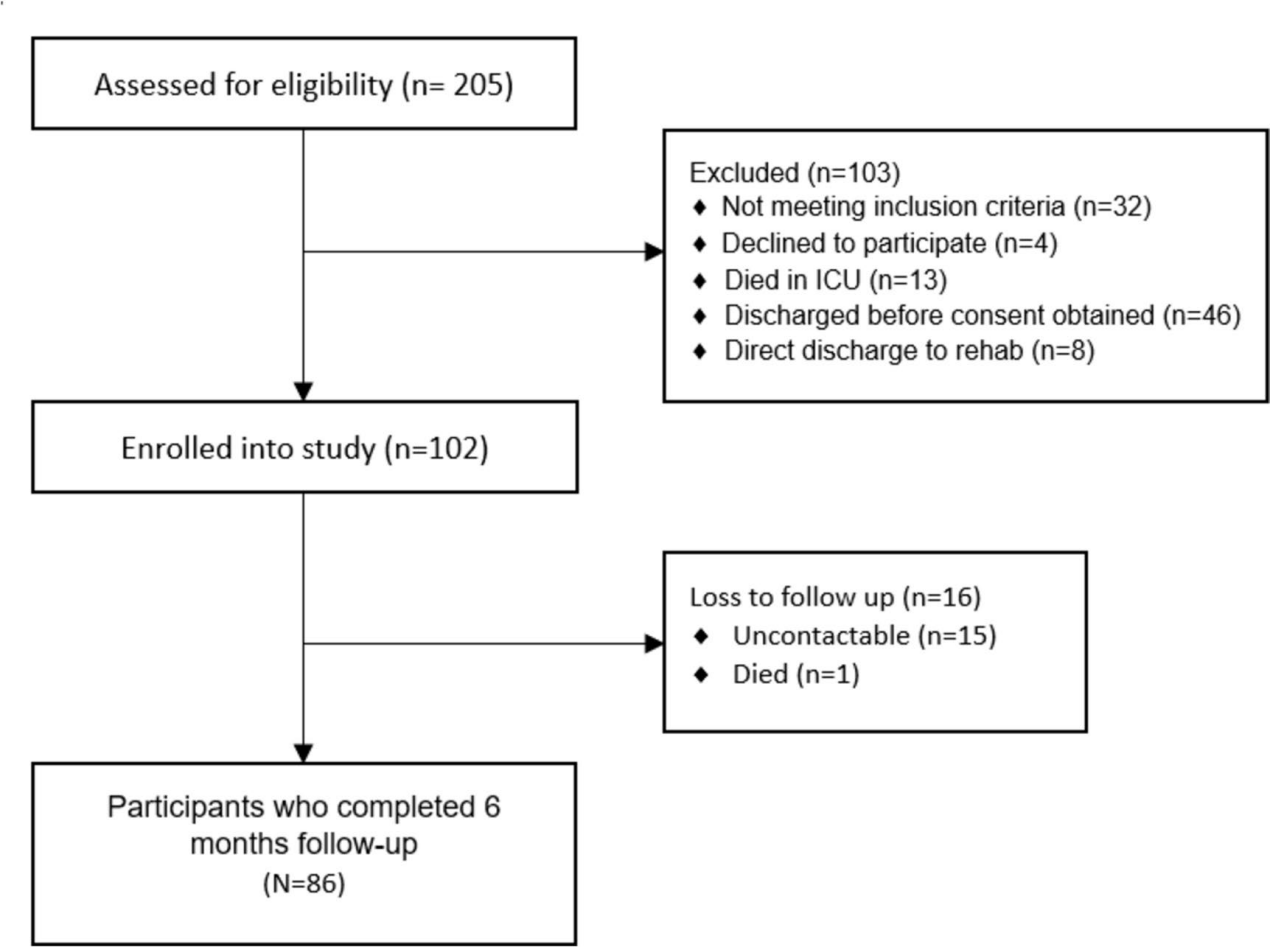
Flow of patients through the study

A total of 102 participants meeting the inclusion criteria were enrolled into this study. Figure 1 shows the flow of patients through the study, including those that were loss to follow up. Data was analysed for the 86 participants who completed the 6-month follow-up. No differences were observed between participants who were a loss to follow up and those that had completed the 6-month follow up.

Table 1 gives an overview of sociodemographic and RTW characteristics for the total participants who completed 6-month follow up, RTW and no RTW at 6-month follow up. The mean age was 39.6 (10.4) years and participants were predominately male (82.6%). Thirty-three of the 60 participants who reported school as their highest education level had completed grade 12 schooling. Two thirds (66.3%) of participants occupied low skill level jobs. The 39 participants (45.3%) who were injured on duty had their medical care funded by the workmen’s compensation fund (WCA). Forty six participants (53.5%) have either applied for or received some form of grant within the 6-month period. The top three causes of injury were road-related injuries which occurred among 45 participants (52.3%), followed by gunshot wounds (GSWs) (14%) and then burns (12.8%). ICU-AW was identified in 17 participants (19.8%). None of the participants had reported any disabilities prior to hospital admission.

**Table 1:** Sociodemographic and return to work (RTW) characteristics of total participants who completed 6-month follow up, RTW and no RTW at 6-month follow up

Variable	Completed 6-month follow-up (Total) (N = 86)	RTW at 6 months (n = 64)	No RTW at 6 months (n = 22)	*p* value*
Age, mean (SD)	39.6 (10.4)	39.7 (10.3)	39.6 (10.8)	0.961
Sex, n (%)				
Male	71 (82.6)	53 (82.8)	18 (81.8)	1.000
Female	15 (17.4)	11 (17.2)	4 (18.2)	
Highest education level, n (%)				
School	60 (69.8)	42 (65.6)	18 (81.8)	**0.187**
Tertiary	26 (30.2)	22 (34.4)	4 (18.2)	
Occupation skill level, n (%)				
High skill level	29 (33.7)	25 (39.1)	5 (22.7)	0.297
Low skill level	57 (66.3)	39 (60.9)	17 (77.3)	
Pre-existing co-morbidity, n (%)	17 (19.8)	14 (21.9)	3 (13.6)	0.541
Funding type, n (%)				
Medical aid	47 (54.7)	38 (59.4)	7 (31.8)	0.029*
Workmen’s compensation fund	39 (45.3)	26 (40.6)	15 (68.2)	
Applied/receiving grant, n (%)				
Workmen's compensation	40 (46.5)	26 (40.6)	14 (63.6)	0.003*
Disability	5 (5.8)	1 (1.6)	4 (18.2)	
Road accident fund	1 (1.2)	1 (1.6)	0 (0)	
No grant	40 (46.5)	36 (56.3)	4 (18.2)	
Incident, n (%)				
Motor vehicle crash	31 (36)	23 (35.9)	8 (36.4)	
Motor bike crash	13 (15.1)	12 (18.8)	1 (4.5)	
Pedestrian vehicle crash	1 (1.2)	1 (1.6)	0 (0)	
Stab	2 (2.3)	2 (3.1)	0 (0)	
Gunshot	12 (14)	9 (14.1)	3 (13.6)	
Crush	6 (7)	2 (3.1)	4 (18.2)	
Fall	3 (3.5)	3 (4.7)	0 (0)	
Burn	11 (12.8)	8 (12.5)	3 (13.6)	
Machine	5 (5.8)	3 (4.7)	2 (9.1)	
Aircraft	1 (1.2)	0 (0)	1 (4.5)	
Sport	1 (1.2)	1 (1.6)	0 (0)	
Mechanism of injury, n (%)				
Blunt	58 (67.4)	44 (68.8)	14 (63.6)	0.742
Penetrating	18 (20.9)	12 (18.8)	6 (27.3)	
Burn	10 (11.6)	8 (17.5)	2 (9.1)	
Discharge destination, n (%)				
Home	82 (95.3)	63 (98.4)	19 (86.4)	0.050*
Rehab facility	4 (4.7)	1 (1.6)	3 (13.6)	
Clinical factors				
HLOS, median (IQR)	21 (3–140)	17.5 (3–140)	34.5 (4–65)	0.027*
ICU LOS, median (IQR)	12 (2–114)	10 (2–114)	13.5 (6–49)	**0.102**
Required ventilation, n (%)	39 (45.3)	26 (40.6)	11 (50)	0.333
ISS, median (IQR)	17 (5–43)	17 (8–38)	18 (5–43)	0.757
Major trauma, ISS > 12, n (%)	61 (70.9)	50 (78.1)	11 (50)	0.016*
GCS, median (IQR)	15 (3–15)	15 (3–15)	15 (3–15)	0.698
RTS, median (IQR)	7.8 (1.5–7.8)	7.8 (1.5–7.8)	7.8 (5.7–7.8)	0.680
CPAx, median (IQR)	50 (17–50)	50 (17–50)	45 (24–50)	0.005*
ICU- AW, MRC-SS < 48, n (%)	17 (19.8)	8 (12.5)	9 (40.9)	0.004*

*SD* standard deviation, *n* number, *%* percentage, *IQR* interquartile range, *Highest education level, school* grade 1–12, *tertiary* university/college/equivalent, *HLOS* hospital length of stay in days, *ICU LOS* intensive care unit length of stay in days, *ISS* Injury Severity Score, *RTS* Revised Trauma Score, *CPAx* Chelsea Critical Care Physical assessment tool, *ICU-AW* intensive care unit acquired weakness, *MRC-SS* Medical Research Council sum score

*Statistical significance (*p* < 0.05) between those that RTW vs no RTW at 6-months follow up. The variables with bold *p* values (*p* < 0.25) were included in the univariate and multivariable analysis

**Fig. 2:**
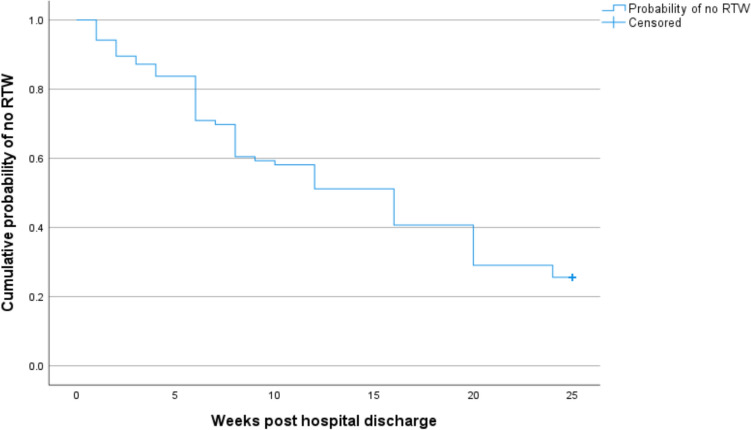
Kaplan–Meier estimate of the cumulative probability of participants not returning to work (RTW)

Figure 2 presents the Kaplan–Meier estimate of the cumulative probability of participants that did not RTW. The median time of RTW (*N* = 86) was 16 weeks. After 24 weeks the cumulative probability of not RTW was 25.6%.

### Patterns of RTW

At six months post hospital discharge, 64 participants (74.4%) had RTW. Changes in work duties occurred among 25 participants who RTW as seen in Table 2 . All 25 participants reported this change was due to physical limitations. Twenty-three of those participants also reported a decrease in work hours. Seven out of the 25 participants who had RTW had to stop work altogether (failed RTW) due to health-related reasons. None of the participants reported mental or cognitive limitations as being a reason for their change in work duties. Health-related reasons were also reported by 18 of the 22 participants who were not able to RTW in any capacity.

**Table 2:** Changes in return to work (RTW) versus no RTW at 6 months follow-up

Variable	Total (N = 86)	RTW (n = 64)	No RTW (n = 22)
Work situation at 6 months, n (%)			
Full time	52 (60.5)	52 (81.3)	N/A
Part time	5 (5.8)	5 (7.8)	N/A
Paid sick leave	14 (16.3)	5 (7.8)*	9 (40.9)
Unpaid sick leave	6 (7)	0 (0)	6 (27.3)
Temporarily laid off	1 (1.2)	0 (0)	1 (4.5)
Unemployed looking for work	2 (2.3)	1 (1.6)*	1 (4.5)
Unemployed not looking for work	1 (1.2)	0 (0)	1 (4.5)
Disabled	5 (5.8)	1 (1.6)*	4 (18.2)
Change in occupation, n (%)	6 (7)	6 (9.4)	N/A
Change in work duties, n (%)	25 (29.1)	25 (39.1)	N/A
Average number of hours worked prior to hospitalization, mean, (SD)	46 (12)	46 (11)	47 (16)
Average number of hours worked post hospitalization, mean, (SD)	40 (14)	40 (14)	N/A
Primary income earner, n (%)			
Pre-hospitalization	60 (69.8)	46 (71.9)	14 (63.6)
Post-hospitalization	59 (68.6)	46 (71.9)	13 (59.1)
Earnings post hospitalization, n (%)			
75–100% as before	48 (55.8)	54 (84)	4 (18.2)
50% to 74% of earnings	9 (10.5)	5 (7.8)	4 (18.2)
Less than 50 of earnings before	2 (2.3)	1 (1.6)	1 (4.5)
More than before	2 (2.3)	2 (3.1)	0 (0)
Not receiving an income	15 (17.4)	2 (3.1)*	13 (59.1)

*SD* standard deviation, *n* number, *%* percentage, *N/A* not applicable

*Failed RTW i.e. participants who RTW but were unable to sustain RTW

All participants were working prior to being admitted to hospital, of which 81 participants (94.2%) were working full time and five participants (5.8%) working part time. Full-time employment dropped by a third to 60.5% with part-time employment remaining unchanged at 5.8% post hospitalisation. Following hospitalisation, health-related reasons were stated by all the newly unemployed participants.

### Factors associated with RTW outcomes

The characteristics of participants who RTW and who did not RTW are shown in Table 1. No significant differences were identified in age, sex, presence of co-morbidities or occupation skill level for participants who RTW compared to those that did not RTW. Mechanism of injury, RTS, GCS and whether a participant required ventilation were also not significantly associated with the RTW outcome. Many of the variables that were initially considered to be significant in the univariate analysis were found to no longer be significant after adjusting for confounding of other variables in the multivariable analysis. Table 3 presents the findings from the univariate and multivariable Cox proportional hazards regression analysis. In the multivariable analysis, CPAx scores (*p* = 0.007), and whether grants were applied/received (*p* = 0.002) were the only factors that were associated with the RTW outcome (Table 3). Every point increase in the CPAx score was associated with a 6% better chance of RTW and participants were 2.2 times more likely to RTW if they did not apply/receive any grants compared to those that did. For every additional HLOS day, the probability of RTW decreased by 1.1%.

**Table 3:** Cox proportional hazards regression analysis for factors associated with time to return to work (RTW)

Variable	Univariate analysis		Multivariable analysis			
			Before backward		After backward	
	Crude hazard ratio (95% CI)	*p* value	Adjusted hazard ratio (95% CI)	*p* value	Adjusted hazard ratio (95% CI)	*p* value
Highest Education level						
School	0.65 (0.39–1.10)	0.114	0.81 (0.47–1.42)	0.477		
Tertiary	1		1			
Funding						
WCA	1.72 (1.04–2.85)	0.033	0.96 (0.19–4.70)	0.963		
Medical aid	1		1			
Discharge destination						
Home	1		1			
Rehab facility	4.87 (0.67–35.17)	0.116	2.76 (0.36–20.87)	0.342		
ICU-AW, MRC-SS < 48						
Yes	0.37 (0.17–0.79)	0.010	1.33 (0.56–3.20)	0.512		
No	1		1			
Grant						
No	0.46 (0.28–0.76)	0.003	1.95 (0.41–9.19)	0.395	2.26 (1.35–3.77)	0.002
Yes	1		1		1	
Major trauma, ISS > 12						
Yes	0.57 (0.31–1.04)	0.070	1.24 (0.66–2.35)	0.492		
No	1		1			
HLOS	0.98 (0.97–1.00)	0.070	0.98 (0.95–1.00)	0.105	0.98 (0.97–1.00)	0.061
ICU LOS	0.99 (0.97–1.00)	0.415	1.01 (0.98–1.04)	0.383		
CPAx	1.06 (1.01–1.11)	0.005	1.05 (1.00–1.00)	0.027	1.06 (1.01–1.10)	0.007

*WCA* workmen’s compensation fund, *ICU-AW* intensive care unit acquired weakness, *MRC-SS* Medical Research Council sum score, *ISS* Injury Severity Score, *HLOS* hospital length of stay, *ICU LOS* intensive care unit length of stay, *CPAx* Chelsea Critical Care Physical assessment tool, *CI* confidence interval

## Discussion

Three quarter of patients discharged from a major trauma centre in South Africa had returned to work within six months of discharge. The median RTW time in this cohort was 16 weeks. Notably, after adjusting for confounding variables, only the CPAx scores at unit discharge and whether grants had been applied/received remained significantly associated with RTW outcomes.

Comparing our results with HIC studies such as Australia [43] and the Netherlands [20], we found similar RTW rates among major trauma survivors but observed differences in work duty modifications and the absence of reported mental or cognitive limitations among our participants. While previous studies have highlighted the impact of anxiety and depression on RTW outcomes, our findings may reflect cultural beliefs and the stigma surrounding mental health in South Africa [28]. Furthermore, the lack of specific and sensitive mental health screening tools and the exclusion of severe head injuries may explain our findings.

Our study underscores the importance of physical function at ICU discharge, as measured by CPAx scores, in predicting RTW outcomes among major trauma survivors. Notably, higher physical function at ICU discharge was associated with a greater likelihood of RTW. These findings highlight the potential value of early rehabilitation interventions in ICU, specifically targeting the physical limitations identified using the CPAx tool.

Contrary to expectations based on previous literature [7, 8, 22, 44, 45], we found that participants in physically demanding or low-skill jobs still returned to work despite limitations. This challenges assumptions about the impact of major trauma on employability and suggests that many survivors may choose to RTW in reduced capacities if given the opportunity, reflecting successful recovery and meaningful integration back into the workforce [46, 47].

Demographic characteristics of our study population, such as age and gender distribution, were consistent with previous studies in SA and globally, indicating a uniformity in major trauma populations across different healthcare systems [7, 13, 14, 20, 21, 48]. However, differences in injury mechanisms, particularly the prevalence of GSWs in SA compared to falls in HICs [7, 8, 20], highlight unique contextual factors that may influence RTW outcomes.

While compensation status has been linked to lower RTW rates in other studies [7, 8, 20, 21, 44], our findings suggest a more complex relationship influenced by economic pressures and job security concerns in LMICs. Despite compensation status being associated with RTW outcomes in our study, the relatively high RTW rates among compensated individuals underscore the multifaceted motivations driving RTW decisions in resource-constrained settings.

The implications of major trauma extend beyond individual survivors to their families, employers, funders, and society as a whole. Early identification of patients at risk of not RTW is crucial for targeted rehabilitation interventions, potentially shortening RTW time and improving overall outcomes. From a societal perspective, early RTW may contribute to improved productivity and economic growth.

While our study provides valuable insights into RTW outcomes among major trauma survivors in SA, several limitations warrant consideration. Given that this study was the first of its kind in SA and since SA has no national trauma registry or database to draw from as were used in many international studies coupled with the vast differences of RTW percentages from global studies, we were unable to perform an accurate sample size calculation. Therefore, due to the small sample size, results of our study limit generalisability and should be interpreted with caution. However, these results provide important contextual data which larger studies can draw to inform sample sizes in the future. Only one point in time follow-up was done in a relatively short period of time and therefore important patterns of RTW such as the participants ability to sustain RTW and changes in RTW rates over time were not able to be adequately observed. Even though no sociodemographic differences were seen between participants who completed the study and those that were a loss to follow up, the unobtainable RTW data could potentially have impacted our study findings. Additionally, our study was conducted in a private level 1 trauma centre, which is known to be adequately resourced and follow international guidelines for standard level of care provided. The private sector is mostly funded through individual contributions to medical aid schemes or health insurance and serves approximately 27% of the population. This differs to the public sector facilities, to which many patients are admitted to. The public sector is state funded and serves 71% of the population. These facilities are usually under-resourced and understaffed. Thus, discrepancies may be seen between the two sectors of the health care system in SA. Financial and cultural differences may also be seen within the public and private sector populations. Consequently, our results may not fully represent the broader population and healthcare landscape in SA and cannot be generalised to the general population. Future research is needed to better understand the complexities of RTW following major trauma in SA.

## Conclusion

During a 6-month period post hospital discharge, 74.4% of major trauma survivors had RTW. Many survivors had RTW in a reduced physical capacity and some with salary cuts. This study has highlighted the complex nature of RTW and the socioeconomic burden following major trauma. Our study contributes to the understanding of RTW outcomes among major trauma survivors in SA and highlights the importance of physical function in predicting RTW. This study is the first of its kind in SA and therefore serves as a baseline for future studies. Further research is needed to explore the broader socioeconomic implications of major trauma and to develop targeted interventions aimed at optimising RTW outcomes for survivors.

## Data Availability

No datasets were generated or analysed during the current study.
